# Nutrition and metabolism in burn patients

**DOI:** 10.1186/s41038-017-0076-x

**Published:** 2017-04-17

**Authors:** Audra Clark, Jonathan Imran, Tarik Madni, Steven E. Wolf

**Affiliations:** University of Texas Southwestern Medical Center, 5323 Harry Hines Blvd., Dallas, TX 75390 USA

**Keywords:** Burn, Nutrition, Metabolism, Critical care

## Abstract

Severe burn causes significant metabolic derangements that make nutritional support uniquely important and challenging for burned patients. Burn injury causes a persistent and prolonged hypermetabolic state and increased catabolism that results in increased muscle wasting and cachexia. Metabolic rates of burn patients can surpass twice normal, and failure to fulfill these energy requirements causes impaired wound healing, organ dysfunction, and susceptibility to infection. Adequate assessment and provision of nutritional needs is imperative to care for these patients. There is no consensus regarding the optimal timing, route, amount, and composition of nutritional support for burn patients, but most clinicians advocate for early enteral nutrition with high-carbohydrate formulas.

Nutritional support must be individualized, monitored, and adjusted throughout recovery. Further investigation is needed regarding optimal nutritional support and accurate nutritional endpoints and goals.

## Background

Nutritional support is a critical aspect of the treatment of burn patients. The metabolic rate of these patients can be greater than twice the normal rate, and this response can last for more than a year after the injury [1, 2]. Severe catabolism accompanies the hypermetabolic state and leads to a tremendous loss of lean body mass as well as a decline of host immune function [3]. Significant nutritional support to meet increased energy expenditure is vital for burn patients’ survival. Unfortunately, our knowledge regarding the complicated physiology of nutrition is incomplete and nutritional regimens vary widely between individual centers. Many questions still exist concerning the optimal route, volume, and composition of diet in the burn population. This article will review the current state of nutrition after burn injury.

## Review

### The hypermetabolic state

**Fig. 1:**
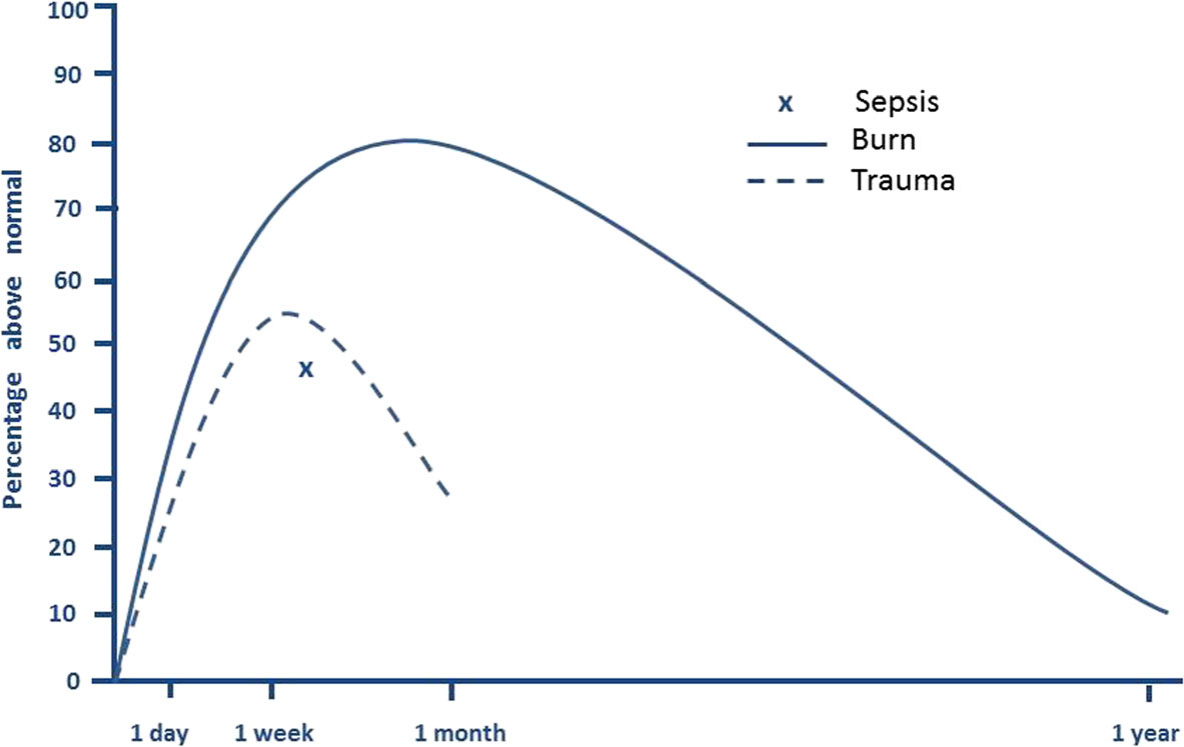
Hypermetabolic response after severe burn, trauma, and sepsis. Adapted from references [5, 6, 123, 124]

Severe burns cause a profound pathophysiological stress response and a radically increased metabolic rate that can persist for years after injury. Trauma and sepsis also result in hypermetabolism, although to a much lesser degree and for a significantly shorter duration (Fig. 1). Immediately after severe injury, patients have a period of decreased metabolism and reduced tissue perfusion known as the “ebb” phase. Soon after, they enter the phase of hypermetabolic rates and hyperdynamic circulation, referred to as the “flow” state [4]. This hypermetabolic state reflects an increase in whole-body oxygen consumption, and a patient is usually considered hypermetabolic when resting energy expenditure (REE) is more than 10% above normal [5]. In the acute postburn injury phase, patients with a burn that covers greater than 40% of total body surface area (TBSA) have a REE between 40 and 100% above normal [6, 7]. It is important to mitigate this stress response and support the significantly increased metabolic needs of the patient as unchecked hypermetabolism results in an enormous loss of lean muscle mass, immune compromise, and delayed wound healing.

Hypermetabolism after burn is very complicated and not yet fully understood. The underlying mechanisms of this vast metabolic, hormonal, and inflammatory dysregulation are still being actively investigated. At a cellular level, increased whole-body oxygen consumption supports greater adenosine triphosphate (ATP) turnover and thermogenesis. ATP-consuming reactions represent an estimated 57% of the hypermetabolic response to burns, including ATP turnover for protein synthesis, ATP production for hepatic gluconeogenesis, and the cycling of glucose and fatty acids [8]. Because ATP turnover does not completely account for burn-induced hypermetabolism, it implies that mitochondrial oxygen consumption exceeds ATP production after severe burn. This likely occurs via the uncoupling of mitochondrial respiration from ADP phosphorylation resulting in heat production [5]. This theory is supported by the recent finding that uncoupling protein 1 (UCP1), a mitochondrial transmembrane protein and a principal mediator of thermogenesis, is much more abundant in the adipose tissue of burn patients compared to healthy individuals [9, 10].

Several studies implicate catecholamines as a primary mediator of hypermetabolism [11, 12]. The elevation of catabolic hormones epinephrine, cortisol, and glucagon lead to the inhibition of protein synthesis and lipogenesis [13]. Protein breakdown becomes a necessary and large source of energy, and skeletal muscle cachexia results from a long-lasting imbalance between protein synthesis and breakdown. The dysregulation of skeletal muscle kinetics lasts a year or more after severe burn, and reduced lean body mass is reported in patients up to 3 years after injury [14–16].

Adequate and prompt nutrition is extremely important for preventing numerous complications, although nutrition has a complex relationship with the hypermetabolic state. In animal models, early nutrition, usually defined as within 24 h of injury, has been shown to actually mitigate burn-induced hypercatabolism and hypermetabolism, although data in humans have not borne this out [17, 18]. A study by Hart et al. compared burned children who had early aggressive feeding and wound excision to burned children who had delay to this treatment, with the authors expecting to find that early surgical treatment and aggressive enteral nutritional support would limit the hypermetabolic response to burn. Surprisingly, they found that the late treatment cohort had significantly lower energy expenditure than the early treatment group. Furthermore, the children with delayed nutrition and surgical excision had a significant increase in their energy expenditure after the initiation of therapy. The authors concluded that excision and aggressive feeding are requisite for the full expression of burn-induced hypermetabolism. Muscle protein catabolism, on the other hand, was significantly decreased in the patients who received early treatment [19]. Burn patients are in a catabolic state that can lead to significant weight loss and associated complications. A 10% loss of total body mass leads to immune dysfunction, 20% to impaired wound healing, 30% to severe infections, and 40% to mortality [20]. Early enteral feeding does result in improved muscle mass maintenance, the modulation of stress hormone levels, improved gut mucosal integrity, improved wound healing, decreased risk of Curling ulcer formation, and shorter intensive care unit stay and is therefore universally recommended despite its link to the hypermetabolic state [21, 22].

Many other therapies to ameliorate burn-induced hypermetabolism have been investigated. Environmental management with the warming of patients’ rooms and occlusive wound dressings attenuate the hypermetabolic response because burn patients have lost their skin barrier and therefore need to produce more heat to maintain thermal neutrality. Early wound excision and grafting have led to improvements in mortality, decreased exudative protein loss, lower risk of burn wound infection, and decreased muscle catabolism [19, 23]. This may be due to a decrease in the levels of circulating inflammatory cytokines such as interleukin (IL)-6, IL-8, C3 complement, and tumor necrosis factor (TNF)-α [24].

Several proven pharmacologic methods can be used to decrease the hypermetabolic response to burn. Beta-adrenergic receptor blockade, usually with propranolol, lowers the heart rate and metabolic rate in patients with severe burns [25–27]. Recently, propranolol treatment for 1-year postburn was shown to improve peripheral lean body mass accumulation [28]. Oxandrolone, a synthetic androgen, has been shown to blunt hypermetabolism, improve bone mineral content and density, and increase the accretion of lean body mass in children with severe burn [29–32]. Recombinant human growth hormone (rHGH) has been found to reduce hypermetabolism and improve lean body mass accretion after burn, but its use has been limited because of two multicenter trials showing that growth hormone therapy increased mortality in critically ill adults [33–35]. More research is needed regarding the efficacy and safety of rHGH use in burn patients.

### Timing of nutritional support

Time to treatment, including time to nutrition, is an important factor for patient outcome after severe burn. Substantial intestinal mucosal damage and increased bacterial translocation occur after burn and result in decreased absorption of nutrients [36]. Because of this, nutritional support should ideally be initiated within 24 h of injury via an enteral route [2, 19]. In animal models, early enteral feeding has been shown to significantly attenuate the hypermetabolic response after severe burn. Mochizuki et al. demonstrated that guinea pigs who were continuously fed enterally starting at 2 h after burn had a significant decrease in metabolic rate at 2 weeks after burn compared to animals whose nutrition was initiated 3 days after burn [17]. This improvement of the hypermetabolic response has not borne out in human studies; however, early enteral nutrition (EN) has been shown to decrease circulating catecholamines, cortisol, and glucagon and preserve intestinal mucosal integrity, motility, and blood flow [18, 37–40]. Early enteral feeding in humans has also shown to result in improved muscle mass maintenance, improved wound healing, decreased risk of Curling ulcer formation, and shorter intensive care unit stay [21, 22]. Nutrition, both parenteral and enteral, is almost always administered in a continuous fashion. For parenteral nutrition (PN), this is done for logistical reasons, but reasons for continuous feeding are less clear for EN. At the start, enteral feeding is initiated in a continuous and low volume manner with slow titration to the goal volume to insure that the patient can tolerate this regimen. A continuous schedule is usually continued even when the patient is having no issues with tolerance. Continuous enteral feeding is likely a holdover from parenteral schedules and no data have shown the superiority of either schedule, but the data are limited [41]. Normal physiology functions with intermittent feeding usually during daytime hours, and further research is needed to determine if there might be a benefit to intermittent feeding after burn.

### Caloric requirements

The primary goal of nutritional support in burn patients is to fulfill the increased caloric requirements caused by the hypermetabolic state while avoiding overfeeding. Numerous formulas to estimate the caloric needs of burn victims have been developed and used throughout the years [42]. One of the earliest examples is the Curreri formula [43]. It was proposed in 1972 and created by studying 9 patients and computing backwards to approximate the calories that would have been needed to compensate for the patients’ weight loss. The Curreri formula and many other older formulas overestimate current metabolic requirements, and more sophisticated formulas with different variables have been proposed (Table 1) [44]. One study of 46 different formulas for predicting caloric needs in burn patients found that none of them correlated well with the measured energy expenditure in 24 patients [1]. Energy expenditure does fluctuate after burn, and fixed formulas often lead to underfeeding during periods of highest energy utilization and to overfeeding late in the treatment course.

**Table 1:** Common formulas used to calculate caloric needs of burn patients

Adult formulas	Kcal/day	Comments
Harris Benedict	Men: 66.5 + 13.8(weight in kg) + 5(height in cm) − 6.76(age in years) Women: 655 + 9.6(weight in kg) + 1.85(height in cm) − 4.68(age in years)	Estimates basal energy expenditure; can be adjusted by both activity and stress factor, multiply by 1.5 for common burn stress adjustment
Toronto Formula	−4343 + 10.5(TBSA) + 0.23(calorie intake in last 24 h) + 0.84(Harris Benedict estimation without adjustment) + 114(temperature) − 4.5(number of postburn days)	Useful in acute stage of burn care; must be adjusted with changes in monitoring parameters
Davies and Lilijedahl	20(weight in kg) + 70(TBSA)	Overestimates caloric needs for large injuries
Ireton-Jones	Ventilated patient: 1784 − 11 (age in years) + 5 (weight in kg) + (244 if male) + (239 if trauma) + (804 if burn) Non-ventilated patient: 629 − 11 (age in years) + 25 (weight in kg) − (609 if obese)	Complex formula which integrates variables for ventilation and injury status
Curreri	Age 16–59: 25(weight in kg) + 40(TBSA) Age >60: 20(weight in kg) + 65(TBSA)	Often overestimates caloric needs
Pediatric formulas		
Galveston	0–1 year: 2100(body surface area) + 1000(body surface area × TBSA) 1–11 year: 1800(body surface area) + 1300(body surface area × TBSA) 12–18 years: 1500(body surface area) + 1500(body surface area × TBSA)	Focuses on maintaining body weight
Curreri junior	<1 year: recommended dietary allowance + 15(TBSA) 1–3 years: recommended dietary allowance + 25(TBSA) 4–15 years: recommended dietary allowance + 40(TBSA)	Commonly overestimates caloric needs

*TBSA* total body surface area

Indirect calorimetry (IC) is the current gold standard for the measurement of energy expenditure, but it is not practical to perform on a routine basis. IC machines measure the volume of expired gas and the inhaled and exhaled concentrations of oxygen and carbon dioxide via tight-fitting face masks or ventilators, allowing for the calculation of oxygen consumption (VO_2_) and carbon dioxide production (VCO_2_), and therefore metabolic rate [45]. IC can also detect underfeeding or overfeeding by calculation of the respiratory quotient (RQ), which is the ratio of carbon dioxide produced to oxygen consumed (VCO_2_/VO_2_) [42]. This ratio is affected by the body’s metabolism of specific substrates. In unstressed starvation, fat is utilized as a major energy source which produces an RQ of <0.7. The normal metabolism of mixed substrates yields an RQ of around 0.75–0.90. Overfeeding is typified by the synthesis of fat from carbohydrate resulting in an RQ of >1.0. This explains one feared complication of overfeeding: difficultly weaning from ventilatory support [46]. Despite this concern, one study found that high-carbohydrate diets in a group of pediatric burn patients led to decreased muscle wasting and did not result in RQs over 1.05 or any respiratory complications [47].

### Substrates

**Fig. 2:**
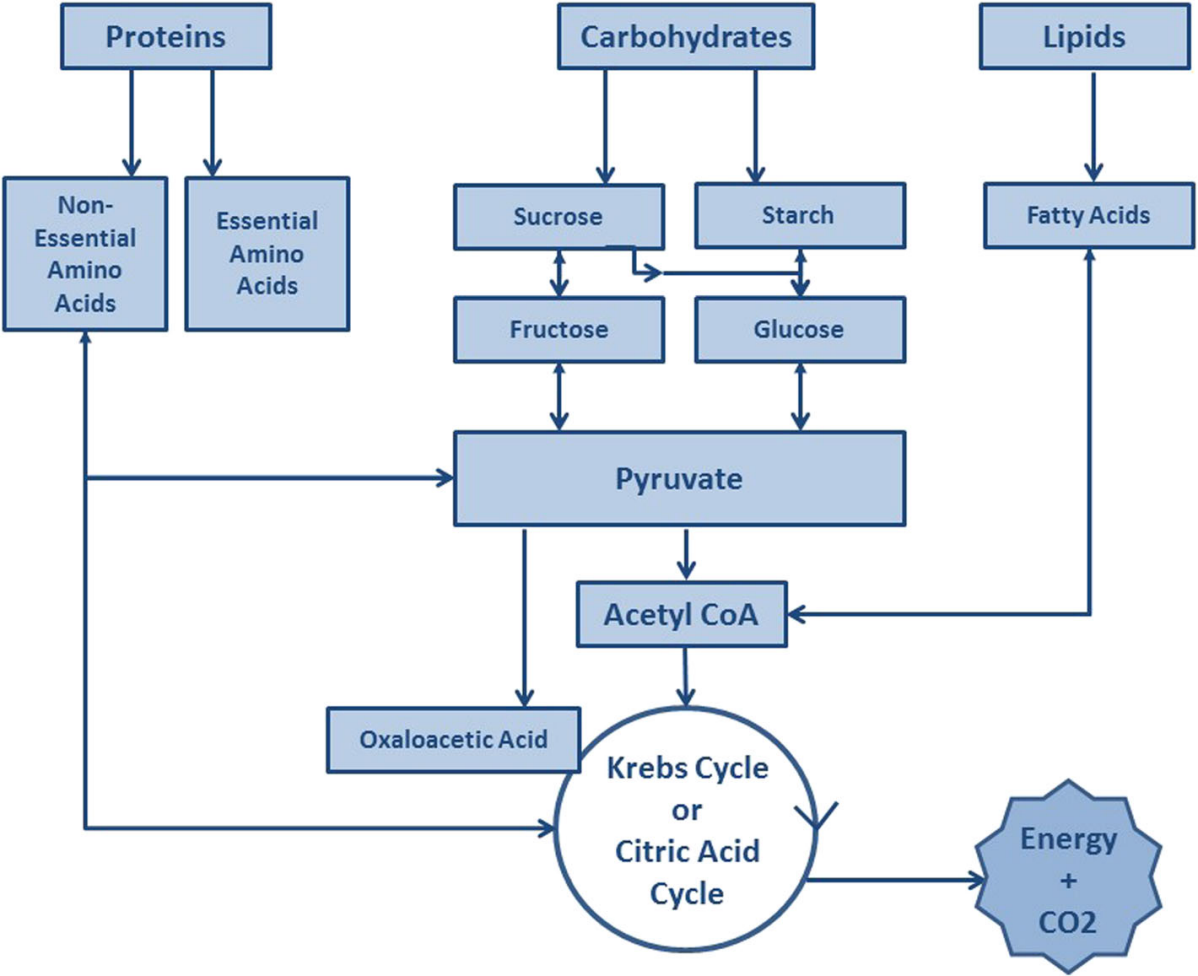
Metabolism of protein, carbohydrates, and lipids

The metabolic process involves the creation and degradation of many products necessary for biological processes. Metabolism of three macronutrients—carbohydrates, proteins, and lipids—provide energy via different pathways (Fig. 2).

#### Carbohydrates

Carbohydrates are the favored energy source for burn patients as high-carbohydrate diets promote wound healing and impart a protein-sparing effect. A randomized study of 14 severely burned children found that those receiving a high-carbohydrate diet (in comparison to a high-fat diet) had significantly less muscle protein degradation [48]. This makes carbohydrates an extremely important part of the burn patient’s diet; however, there is a maximum rate at which glucose can be oxidized and used in severely burned patients (7 g/kg/day) [49, 50]. This rate can be less than the caloric amount needed to prevent lean body mass loss, meaning severely burned patients may have greater glucose needs than can be safely given. If glucose is given in excess of what can be utilized, it leads to hyperglycemia, the conversion of glucose to fat, glucosuria, dehydration, and respiratory problems [51].

The hormonal environment of stress and acute injury causes some level of insulin resistance, and many patients benefit from supplemental insulin to maintain satisfactory blood sugars. Insulin therapy also promotes muscle protein synthesis and wound healing [52]. Studies have found that severely burned patients who received insulin infusions, in conjunction with a high-carbohydrate, high-protein diet, have improved donor site healing, lean body mass, bone mineral density, and decreased length of stay [53, 54]. Hypoglycemia is a serious side effect of insulin therapy, and patients must be monitored closely to avoid this complication.

#### Fat

Fat is a required nutrient to prevent essential fatty acid deficiency, but it is recommended only in limited amounts [13]. After burn, lipolysis is suppressed and the utilization of lipids for energy is decreased. The increased beta-oxidation of fat provides fuel during the hypermetabolic state; however, only 30% of the free fatty acids are degraded and the rest go through reesterification and accumulate in the liver. Additionally, multiple studies suggest that increased fat intake adversely affects immune function [55, 56]. Because of these effects, many authorities recommend very low-fat diets (<15% of total calories) in burn patients where no more than 15% of total calories come from lipids. Multiple low-fat enteral formulas have been created for this purpose, and for patients receiving short-term (<10 days) PN, many clinicians forego lipid emulsions.

In addition to the amount of fat, the composition of administered fat must be considered. The most commonly used formulas contain omega-6 fatty acids such as linoleic acid, which are processed via the synthesis of arachidonic acid, a precursor of proinflammatory cytokines (e.g., prostaglandin E_2_). Lipids that contain a high percentage of omega-3 fatty acids are metabolized without promoting proinflammatory molecules and have been linked to enhanced immune response, reduced hyperglycemia, and improved outcomes [57, 58]. Because of this, omega-3 fatty acids are a major component of “immune-enhancing diets.” Most enteral formulas have an omega 6:3 ratio between 2.5:1 and 6:1 while the immune-enhancing diets have an omega 6:3 ratio closer to 1:1. The ideal composition and amount of fat in nutritional support for burn patients remains a topic of controversy and warrants further investigation.

#### Protein

Proteolysis is greatly increased after severe burn and can exceed a half pound of skeletal muscle daily [59]. Protein supplementation is needed to meet ongoing demands and supply substrate for wound healing, immune function, and to minimize the loss of lean body mass. Protein is used as an energy source when calories are limited; however, the opposite is not true. Giving excess calories will not lead to increased protein synthesis or retention, but rather lead to overfeeding.

Supplying supranormal doses of protein does not reduce the catabolism of endogenous protein stores, but it does facilitate protein synthesis and reduces negative nitrogen balance [60]. Currently, protein requirements are estimated as 1.5–2.0 g/kg/day for burned adults and 2.5–4.0 g/kg/day for burned children. Non-protein calorie to nitrogen ratio should be maintained between 150:1 for smaller burns and 100:1 for larger burns [61]. Even at these high rates of replacement, most burn patients will experience some loss of muscle protein due to the hormonal and proinflammatory response to burn injury.

Several amino acids are important and play unique roles in recovery after burn. Skeletal muscle and organ efflux of glutamine, alanine, and arginine are increased after burn. These amino acids are important for transport and help supply energy to the liver and healing wounds [62]. Glutamine directly provides fuel for lymphocytes and enterocytes and is essential for maintaining small bowel integrity and preserving gut-associated immune function [63, 64]. Glutamine also provides some level of cellular protection after stress, as it increases the production of heat shock proteins and it is a precursor of glutathione, a critical antioxidant [64–66]. Glutamine is rapidly exhausted from muscle and serum after burn injury, and administration of 25 g/kg/day of glutamine has been found to reduce mortality and length of hospitalization in burn patients [67, 68]. Arginine is another important amino acid because it stimulates T lymphocytes, augments natural killer cell performance, and accelerates nitric oxide synthesis, which improves resistance to infection [69, 70]. The supplementation of arginine in burn patients has led to improvement in wound healing and immune responsiveness [70–72]. Despite some promising results in the burn population, data from critically ill nonburn patients suggest that arginine could potentially be harmful [73]. The current data is insufficient to definitively recommend its use, and further study is warranted.

### Vitamins and trace elements

The metabolism of numerous “micronutrients” (vitamins and trace elements) is beneficial after burn as they are important in immunity and wound healing. Severe burn leads to an intense oxidative stress, which combined with the substantial inflammatory response, adds to the depletion of the endogenous antioxidant defenses, which are highly dependent on micronutrients [74, 75]. Decreased levels of vitamins A, C, and D and Fe, Cu, Se, and Zn have been found to negatively impact wound healing and skeletal and immune function [76–78]. Vitamin A decreases time of wound healing via increased epithelial growth, and vitamin C aids collagen creation and cross-linking [79]. Vitamin D contributes to bone density and is deficient after burn, but its exact role and optimal dose after severe burn remains unclear. Pediatric burn patients can suffer significant dysfunction of their calcium and vitamin D homeostasis for a number of reasons. Children with severe burn have increased bone resorption, osteoblast apoptosis, and urinary calcium wasting. Additionally, burned skin is not able to manufacture normal quantities of vitamin D3 leading to further derangements in calcium and vitamin D levels. A study of pediatric burn patients found that supplementation with a multivitamin containing 400 IU of vitamin D2 did not correct vitamin D insufficiency [80–82]. More investigation into therapies to combat calcium and vitamin D deficiency is needed. The trace elements Fe, Cu, Se, and Zn are important for cellular and humoral immunity, but they are lost in large quantities with the exudative burn wound losses [77]. Zn is critical for wound healing, lymphocyte function, DNA replication, and protein synthesis [83]. Fe acts as a cofactor for oxygen-carrying proteins, and Se boosts cell-mediated immunity [75, 84]. Cu is crucial for wound healing and collagen synthesis, and Cu deficiency has been implicated in arrhythmias, decreased immunity, and worse outcomes after burn [85]. Replacement of these micronutrients has been shown to improve the morbidity of severely burned patients (Table 2) [2, 75, 86, 87].

**Table 2:** Vitamin and trace element requirements [125]

Age, years	Vitamin A, IU	Vitamin D, IU	Vitamin E, IU	Vitamin C, IU		Vitamin K, mcg	Folate, mcg	Cu, mg	Fe, mg	Se, mcg	Zn, mg
0–13											
Nonburned	1300–2000	600	6–16	15–50		2–60	65–300	0.2–0.7	0.3–8	15–40	2–8
Burned	2500–5000			250–500			1000^a^	0.8–2.8		60–140	12.5–25
≥13											
Nonburned	200–3000	600	23	75–90		75–120	300–400	0.9	8–18	40–60	8–11
Burned	10,000			1000			1000^a^	4		300–500	25–40

^a^Administered three times weekly

### Routes of nutrition: parenteral vs. enteral

PN was routinely used for burn patients in the 1960s and 1970s, but it has been almost completely replaced by EN [88]. Studies found that PN, alone or in conjunction with EN, is associated with overfeeding, liver dysfunction, decreased immune response, and three-fold increased mortality [89, 90]. PN also appears to increase the secretion of proinflammatory mediators, including TNF, and also can aggravate fatty infiltration of the liver [91, 92]. In addition to these issues, PN has more mechanical and infectious complications of catheters, and PN solutions are significantly more expensive than EN formulas.

EN, in addition to being a safe and cost effective feeding route, has been found to have many advantages. The presence of nutrients within the lumen of the bowel promotes function of the intestinal cells, preserves mucosal architecture and function, stimulates blood supply, decreases bacterial translocation, and improves gut-associated immune function [36, 39]. EN decreases hyperglycemia and hyperosmolarity as it has a “first-pass” hepatic delivery of nutrients [17]. For all of these reasons, EN is the route of choice for severely burned patients. EN can be administered as either gastric or post-pyloric feedings, and both are widely used. Gastric feeding has the advantages of larger diameter tubes, which have less clogging and the ability to give bolus feeds; however, the stomach often develops ileus in the postburn state. Smaller post-pyloric tubes are more prone to clogging and malposition, but they are often more comfortable and post-pyloric feedings can be safely continued even during surgical procedures to sustain caloric goals without an increased risk of aspiration [93]. Despite the strong preference to give nutritional support primarily via the gastrointestinal tract, PN can be used in burned patients in whom EN is contraindicated. Further research is warranted regarding if parenteral supplementation of specific dietary components, such as amino acids alone, would be beneficial. PN and EN are usually given in a continuous fashion.

### Formulas

The earliest formulas for burn patients consisted of milk and eggs, and although these simple mixtures were relatively successful at providing adequate nutrition, they were very high in fat. Numerous commercially prepared enteral formulas have been developed since that time, all with differing amounts of carbohydrates, protein, fats, and micronutrients (Table 3). Glucose is the preferred energy source for burn patients and they should therefore be administered a high-carbohydrate diet [47, 94]. Parenteral formulas usually consist of 25% dextrose, 5% crystalline amino acids, and maintenance electrolytes. This is often supplemented with infusions of 250 mL of 20% lipid emulsions three times a week to meet essential fatty acid needs [95, 96].

**Table 3:** Selected adult enteral nutrition formulas [126]

Formula	Kcal/mL	Carbohydrate, g/L (% calories)	Protein, g/L (% calories)	Fat, g/L (% calories)	Comments
Impact	1.0	130 (53)	56 (22)	28 (25)	IED with arginine, glutamine fiber
Crucial	1.5	89 (36)	63 (25)	45 (39)	IED with arginine, hypertonic
Osmolite	1.06	144 (54)	44 (17)	35 (29)	Inexpensive, isotonic
Glucerna	1.0	96 (34)	42 (17)	54 (49)	Low carbohydrate, for diabetic patients
Nepro	1.8	167 (34)	81 (18)	96 (48)	Concentrated, for patients with renal failure

*IED* immune-enhancing diet

Immune-enhancing diets, or immunonutrition, are nutritional formulas that have been enriched with micronutrients in an effort to improve immune function and wound healing. These formulas gained attention after Gottschlich et al. found that severely burned children given a tube feeding formula containing omega-3 fatty acid, arginine, histidine, and vitamins A and C had significantly fewer wound infections, shorter length of stay, and trended toward improved survival compared to children fed commercially available formulas [97]. This led to the commercial production of similar immune-enhancing diets. Subsequent study of these formulas has shown that they lead to an improvement in neutrophil recruitment, respiratory gas exchange, cardiopulmonary function, mechanical ventilation days, and length of stay in some nonburn populations [98, 99]. Studies in patients with sepsis and pneumonia, however, suggest immune-enhancing diets could have a harmful effect [73, 98]. Little research exists regarding immune-enhancing diets in the burn population. A small study by Saffle et al. found no difference in major outcome variables between the immune-enhancing diet, Impact (Nestle HealthCare, Florham Park, NJ), and a high-protein stress formula, Replete (Nestle HealthCare) [100]. It has been theorized that because of the high volume of feedings given to burn patients, they may receive a satisfactory dose of most immune-enhancing nutrients with the use of conventional diets. A multitude of formulas and numerous methods for calculating nutritional needs are used successfully in the burn population, which suggests that no formula or calculation is perfect, but most are adequate to prevent nutritional complications.

The study of nutrition and metabolism in burn patients is difficult to perform in an exacting and precise method because both the pathophysiology of burn injury and the treatment modalities during the course of burn care are very complex. The effects of differing compositions of nutritional support can easily be confounded by variations in treatment modalities and the complicated pathophysiology of individual burn patients at different stages of their treatment course. A single burn unit takes a very long time to gather data from enough patients which could introduce confounders as other treatment methods advance and change. Multi-institutional trials are also difficult, and any difference in treatment protocols among institutions could overshadow effects of differing nutritional support. A wide range of clinical trials on different nutritional regimens are still being carried out and have not reached convincing consensus on optimal nutrition for burn patients. Physiological/biochemical markers need to be developed or used to assess the potential benefits of these nutrients in parallel to the ongoing evidence-based clinical trials.

### Obesity

The rate of obesity has rapidly grown over the past 30 years in both the USA and worldwide [101]. Approximately two thirds of the US population are overweight, and one third meet the BMI criteria for obese [102]. In the general population, obesity is clearly linked with multiple health problems including diabetes, cardiovascular disease, arthritis, and morbidity [103]. Strangely, overweight and moderately obese patients in surgical and medical intensive care units have been found to have a reduced mortality compared to normal weight patents, despite a higher rate of infections and longer length of stay [104, 105]. Data in the burn population are more limited. A study of the National Burn Repository found a higher mortality for patients listed as obese, but the study was limited due to nonstandard data fields in the database, and the term “obese” was not clearly defined [106]. Two small pediatric studies demonstrated longer hospital stays and a greater need for ventilatory support in obese burned children [107, 108].

Obesity has significant physiologic effects, and fat plays an active role in metabolic regulation. Obesity is associated with an elevated secretion of proinflammatory cytokines, including IL-6, TNF-alpha, and C-reactive protein, and obesity is posited to be a state of chronic inflammation [109, 110]. After burn, obese patients may respond with amplified inflammation, increased hypermetabolism, brisker and more severe muscle wasting, and severe insulin resistance [111]. Obese patients also have decreased bioavailability of vitamin D3 compared to non-obese patients which can potentially worsen vitamin D and calcium deficiency after burn in this population [80].

Obesity also makes initial nutritional assessment difficult as obese patients can still be malnourished, and using actual body weight in predictive formulas overestimates energy needs, while ideal body weight underestimates the needs. A few formulas specifically for obese patients have been created but have not been validated. Some clinicians endorse the use of hypocaloric feeding which consists of low-calorie, high-protein diets with the goal of maintaining lean body mass while promoting weight loss and glycemic control [112]. A few small trials in nonburn patients found that patients on a hypocaloric diet had reduced mortality, ventilator dependence, and length of stay [113, 114]. Data remain very limited in nonburn patients and nonexistent in the burn population, and more studies will need to be done before this can be recommended.

### Monitoring of nutritional support

It is challenging to objectively assess the success of nutritional support of a burn patient, as the true endpoint of therapy is global and cannot be measured by one variable. The overall goal of therapy is to reestablish normal body composition and metabolic equilibrium, and commonly measured variables include body weight, nitrogen balance, imaging of lean body mass, and measurement of serum proteins. Functional measures such as exercise tolerance have also been proposed as a possible metric.

Body weight is a tempting measure of nutritional status as it is easy to obtain and is useful in the general population; however, it can be very misleading in burn patients. The initial fluid resuscitation after severe burn routinely adds 10–20 kg or more of body weight, and although this will eventually lead to diuresis, the time course is unpredictable [115]. Additional fluid shifts occur with infections, ventilator support, and hypoproteinemia, making body weight a very unreliable gauge of nutrition in this population. Patients can have increased total body water for weeks after the burn, which can mask the loss of lean body mass that has certainly occurred [116]. A study of severely burned children found that increasing caloric intake to maintain weight resulted in increased fat mass instead of improved lean body mass [48]. Long-term trends are valuable, and weight should be monitored, especially during the rehabilitation phase.

Providing adequate protein intake is an extremely important part of nutritional support after burn. Nitrogen is a fundamental component of amino acids, and as such, the measurement of nitrogen inputs and losses can be used to study protein metabolism. A positive nitrogen balance is associated with periods of growth as it represents an increase in the total body amount of protein, while negative nitrogen balance occurs with burns, trauma, and periods of fasting. Measurement requires accurate urine collection for determination of urea nitrogen (UUN) as well as documentation of dietary nitrogen intake [117]. Nitrogen balance for burn patients can be approximated with the following formula:

$$ \mathrm{Nitrogen}\ \mathrm{balance} = \mathrm{Nitrogen}\ \mathrm{in}\mathrm{take}\ \mathrm{in}\ 24\ \mathrm{h}\ \hbox{--} \left[1.25\times \left(\mathrm{UUN} + 4\right)\right] $$

Errors in the calculation can come from the two constants. To approximate total urinary nitrogen, 4 g/dL is added to UUN, but total urinary nitrogen may surpass this value in burn patients, leading to an underestimation of nitrogen loss [118, 119]. To account for substantial loss of protein-rich exudates from burn wounds, estimated total urinary nitrogen is multiplied by 1.25, which can similarly underestimate nitrogen losses.

Measurement of serum proteins such as albumin and prealbumin can be utilized to assess nutritional status, but they also have limitations. Metabolic pathways are shifted away from maintenance of these proteins after burn injury, and serum albumin levels are depressed both acutely and chronically, even with successful nutrition, making it a poor marker [120]. Prealbumin has a short half-life of 2 days which theoretically makes it more responsive to nutritional changes. In reality, the level of prealbumin falls quickly after burn and recovers slowly and may not correlate well with ongoing nutritional status [121]. Protein markers, similar to body weight, should be interpreted in context with the patient’s clinical status and with the overall trend in mind.

A few imaging techniques are now available for nutritional monitoring, although due to availability and cost they are typically used in research only. Bioimpedance analysis is a method to calculate total body water and the body’s fat-free cell mass by measuring the body’s resistance to the passage of electrical currents, although it is unknown how the fluid shifts after burn affects this measurement. Another imaging option is dual x-ray absorptiometry (DEXA) scanning, which can measure bone density and lean body mass.

Graves et al. surveyed 65 burn centers in 2007 regarding their nutritional monitoring practices, and the most commonly used parameters were prealbumin (86% of centers), body weight (75%), calorie count (69%), serum albumin (45.8%), nitrogen balance (54%), and transferrin (16%) [122]. No individual method is universally reliable or applicable for the nutritional monitoring of burn patients, and the overall clinical picture must be incorporated into the assessment.

### Overfeeding

The estimation of the nutritional needs of burn patients can be very difficult, and aggressive nutrition in the early post-injury stage can lead to inadvertent overfeeding as the metabolic rate slows and intestinal absorption improves. Overfeeding carries numerous complications, including difficulty weaning from ventilatory support, fatty liver, azotemia, and hyperglycemia. Overfeeding of carbohydrates leads to fat synthesis, increased carbon dioxide, and an increase in the RQ, which worsens respiratory status and makes liberation from the ventilator more challenging [44]. After burn, the hypermetabolic response leads to the mobilization of all available substrates, and this marked increase of peripheral lipolysis can lead to the development of a fatty liver. Overfeeding, via the parenteral or enteral route, can exacerbate the deposition of fat in the liver parenchyma, and fatty liver has been associated with immune dysfunction and increased mortality [92]. Azotemia can occur due to the large amounts of protein administered to burn patients. This is important as the massive fluid shifts after burn can cause a prerenal kidney injury, and increased blood urea nitrogen can aggravate the stress already placed on the kidney. Patients with azotemia which does not respond to hydration may need a reduced amount of protein in their nutrition and need to be closely monitored for signs of renal failure. Nutritional support should be continued in patients with renal failure, but blood chemistries should be checked regularly as metabolic derangements are common and must be addressed.

The predictive formulas of nutritional needs should be used as guidelines, and patients’ energy requirements should be regularly reassessed. As the acute hypermetabolic phase tapers, the more standard equations and injury/activity factors can be used to avoid overfeeding. Factors such as the changing amount of open wound and physical/occupational therapy activity should be taken into account when estimating nutritional needs.

### Nutrition after discharge

It is important that patients continue to receive adequate nutrition after discharge from the hospital, but data on the optimal diet after the acute postburn phase are virtually nonexistent. Because the hypermetabolic state can persist for over a year after burn injury, increased caloric intake with a high protein component is usually recommended for about a year after discharge. Resistance exercise is also recommended to combat continued loss of muscle mass. Patients should regularly weigh themselves to ensure they are maintaining their weight as instructed by the physician and dietician. Oxandrolone is often continued in the outpatient setting, but no data exist regarding the optimum duration of therapy and further study is needed. Nutritional assessments should be a consistent component of outpatient follow-up for burn patients.

## Conclusions

The delivery of nutritional support is a vital element of burn care, and the main goal is simply to avoid nutritional complications. Effective assessment and management can optimize wound healing and decrease complications and mortality. EN with high-carbohydrate formulas is beneficial, although nutritional support must be individualized, monitored, and adjusted throughout recovery. Accurate nutritional endpoints and goals need to be established and validated before the optimal nutritional regimen can be determined. Basic science analysis of the metabolic changes after burn must be coupled with randomized prospective clinical trials to ascertain the ideal nutritional support for the burn patient.

## Abbreviations

ATP: Adenosine triphosphate
EN: Enteral nutrition
IC: Indirect calorimetry
IL: Interleukin
PN: Parenteral nutrition
REE: Resting energy expenditure
rHGH: Recombinant human growth hormone
RQ: Respiratory quotient
TBSA: Total body surface area
TNF: Tumor necrosis factor
UCP1: Uncoupling protein 1
UUN: Urea nitrogen
